# Supplementary material for: Detection of Biomarkers Relating to Quality and Differentiation of Some Commercially Significant Whole Fish Using Spatially Off-Set Raman Spectroscopy
Source: Molecules. 2020 Aug 19;25(17):3776. doi: 10.3390/molecules25173776 (PMC7503569; doi:10.3390/molecules25173776)

RMIT Classification: Trusted

Detection of biomarkers relating to quality and differentiation of some commercially significant whole fish using Spatially Off-set Raman Spectroscopy

Supplementary materials

Figure S1. Average spectra of each region of Asian seabass

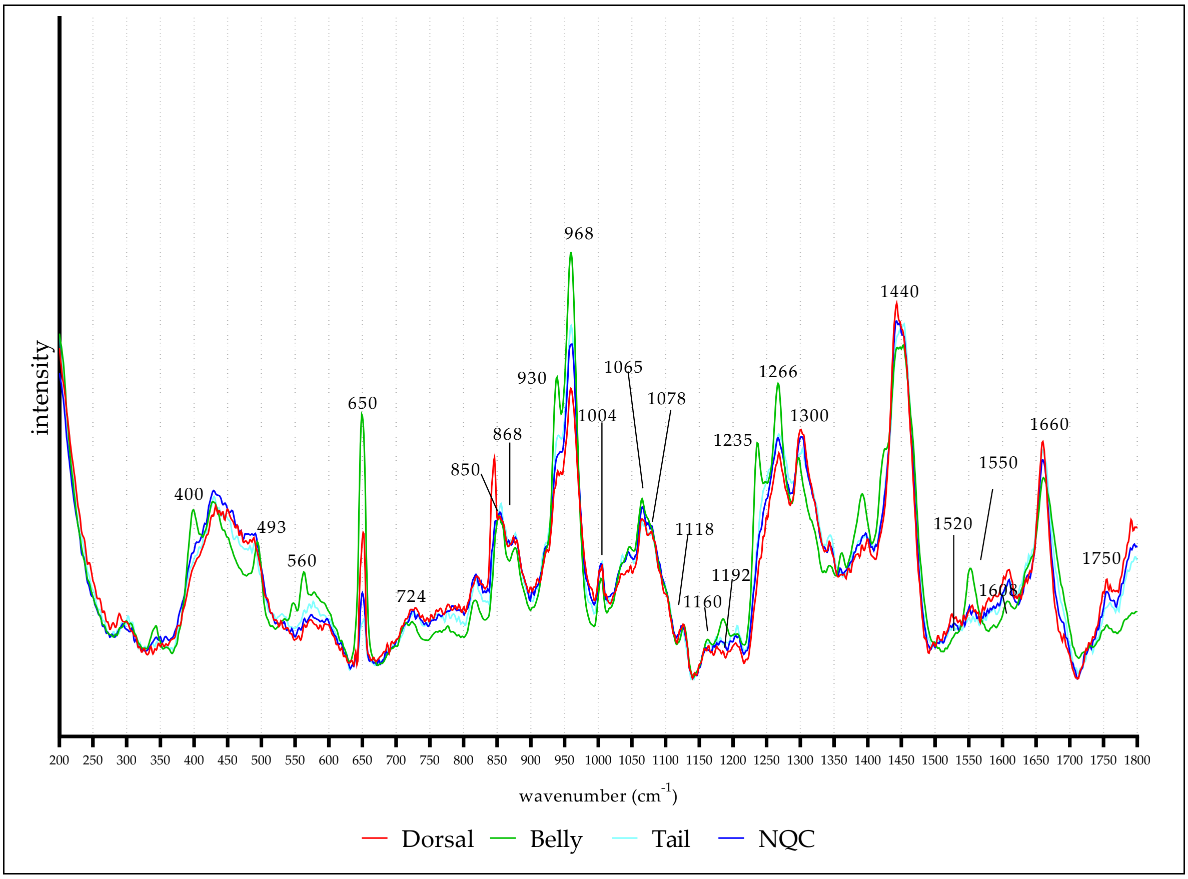

Figure S2. Average spectra of each region of red seabream

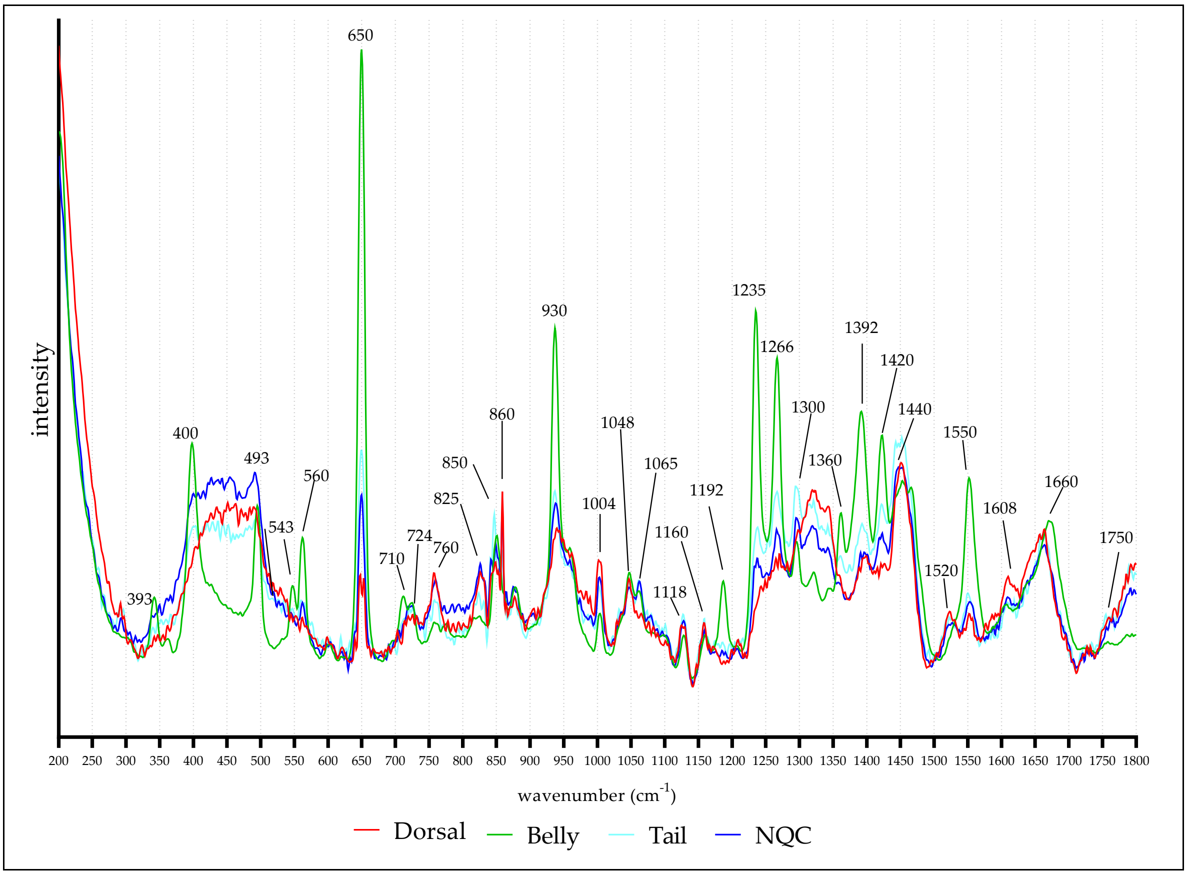

RMIT Classification: Trusted

Figure S3. Average spectra of each region of Atlantic salmon.

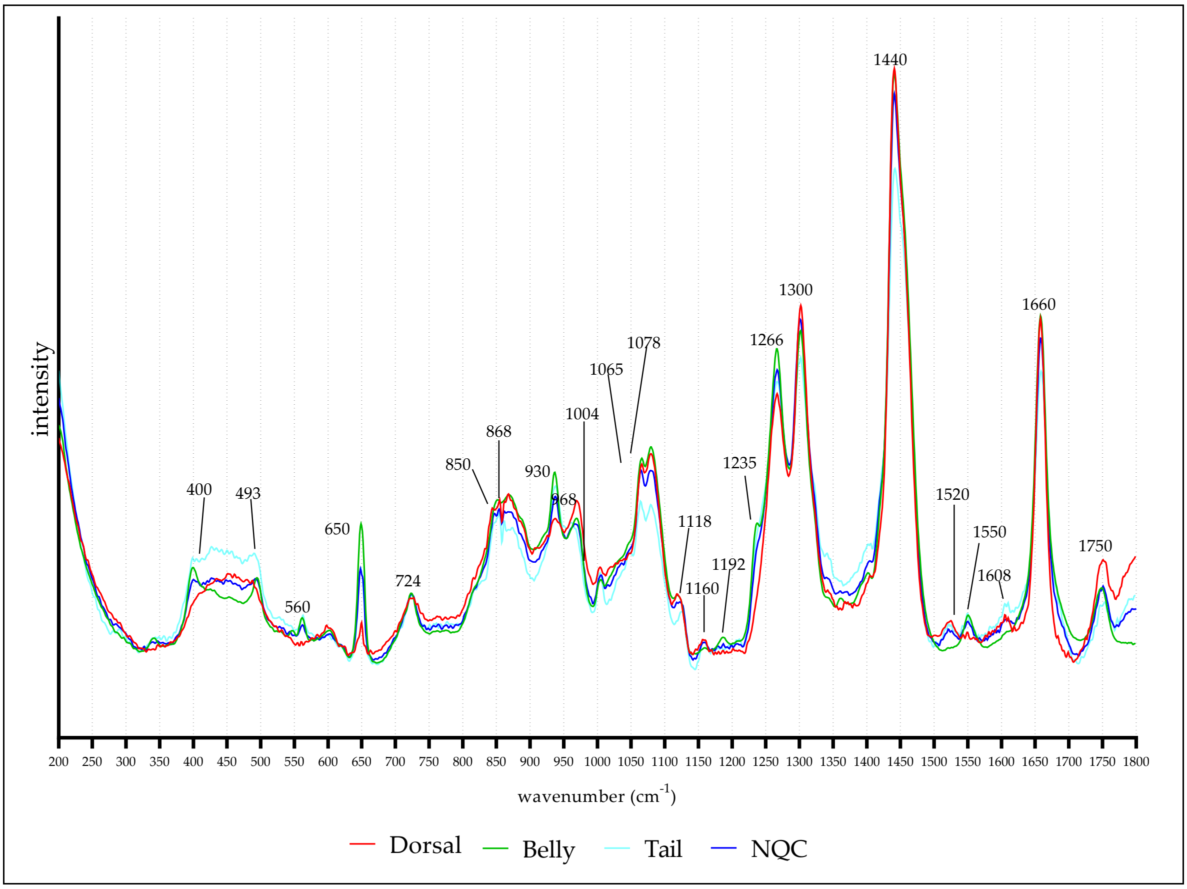

RMIT Classification: Trusted

Figure S4. Average spectra of each region of rainbow trout.

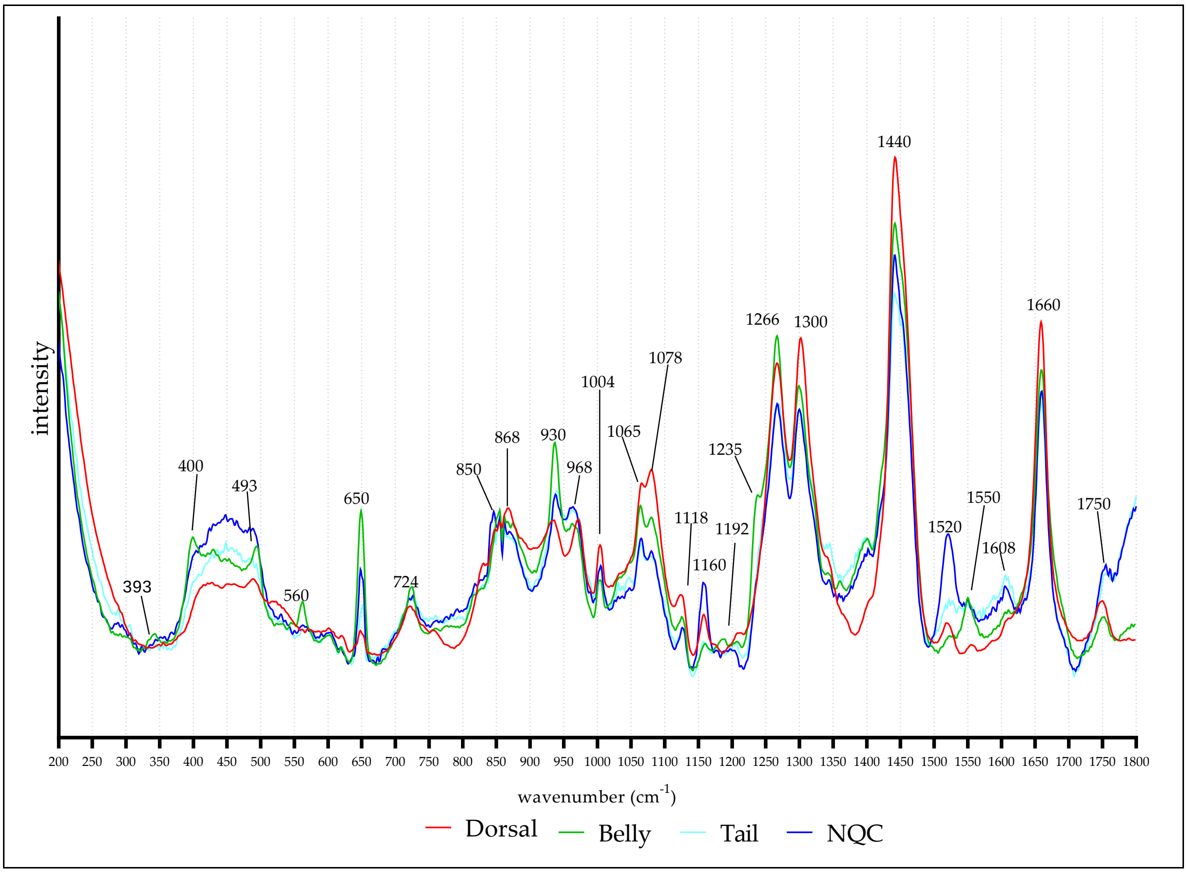

Supplement: Supplementary file 1 [file molecules-25-03776-s001.pdf]
